# Cth2 Protein Mediates Early Adaptation of Yeast Cells to Oxidative Stress Conditions

**DOI:** 10.1371/journal.pone.0148204

**Published:** 2016-01-29

**Authors:** Laia Castells-Roca, Jordi Pijuan, Francisco Ferrezuelo, Gemma Bellí, Enrique Herrero

**Affiliations:** Departament de Ciències Mèdiques Bàsiques, Universitat de Lleida, IRBLleida,Lleida, Spain; Instituto de Biociencias - Universidade de São Paulo, BRAZIL

## Abstract

Cth2 is an mRNA-binding protein that participates in remodeling yeast cell metabolism in iron starvation conditions by promoting decay of the targeted molecules, in order to avoid excess iron consumption. This study shows that in the absence of Cth2 immediate upregulation of expression of several of the iron regulon genes (involved in high affinity iron uptake and intracellular iron redistribution) upon oxidative stress by hydroperoxide is more intense than in wild type conditions where Cth2 is present. The oxidative stress provokes a temporary increase in the levels of Cth2 (itself a member of the iron regulon). In such conditions Cth2 molecules accumulate at P bodies-like structures when the constitutive mRNA decay machinery is compromised. In addition, a null *Δcth2* mutant shows defects, in comparison to *CTH2* wild type cells, in exit from α factor-induced arrest at the G1 stage of the cell cycle when hydroperoxide treatment is applied. The cell cycle defects are rescued in conditions that compromise uptake of external iron into the cytosol. The observations support a role of Cth2 in modulating expression of diverse iron regulon genes, excluding those specifically involved in the reductive branch of the high-affinity transport. This would result in immediate adaptation of the yeast cells to an oxidative stress, by controlling uptake of oxidant-promoting iron cations.

## Introduction

Cellular responses to environmental stresses involve changes in RNA transcript levels, through the modulation of the kinetics of transcription and decay rates of individual mRNA molecules [1]. The yeast *Saccharomyces cerevisiae* has been employed as a model organism in whole-genome studies to demonstrate the importance of differential changes in the decay rate of mRNA molecules during the responses to nutritional, oxidative, osmotic and heat stresses, contributing together with transcription rate changes to reprogramming gene expression [2–6].

One of the stresses that yeast cells may confront is iron (Fe) starvation. Fe is a micronutrient essential for cellular processes such as mitochondrial oxidative phosphorylation, oxygen transport, carbon and lipid metabolism, and DNA replication and repair. However, at high concentrations it is toxic due to Fe-promoted generation of reactive oxygen species (ROS) through the Haber-Weiss cycle, particularly of hydroxyl radicals. As a consequence, intracellular Fe levels must be finely tuned [7]. In *S*. *cerevisiae* Fe uptake occurs through both the low and high affinity systems depending on external Fe availability [8]. The high affinity system is induced under Fe scarcity and it is subjected to transcriptional regulation by the Aft1 factor and its paralog Aft2, with Aft1 playing the main role in the Fe deprivation response [8–11]. Aft1-mediated activation of gene expression involves relocalization of this factor from the cytoplasm to the nucleus. The genes under Aft1/Aft2 control constitute the Fe regulon and encode proteins required for capturing external Fe, its reduction and uptake at the plasma membrane and its mobilization from internal (vacuolar) stores. Transport of external Fe by the high affinity system can occur through a reductive or an alternative non-reductive pathway [8]. In both cases, the Fit1-3 cell wall mannoproteins facilitate Fe movement through the cell wall [12]. Subsequently, the reductive pathway involves reduction of ferric to ferrous ions (these latter ones being potent generators of hydroxyl radicals) and a transport complex formed by the Ftr1 transporter and the Fet3 ferrooxidase. The alternative non-reductive pathway employs siderophore-ferric iron complexes that are transported across the plasma membrane by members of the ARN family of proteins (Arn1–4) [13]. All the mentioned proteins are encoded by components of the Fe regulon.

By contrast, when external Fe concentration is high, yeast cells avoid the prooxidant action of Fe through Yap5-mediated activation of *CCC1*, encoding a Fe transporter at the vacuolar membrane that mediates Fe detoxification by sequestering it at the vacuole, as well as through downregulation of expression of the *FET3/FTR1* genes [14–16].

Other members of the Fe regulon are the *CTH1* and *CTH2* genes. Cth1 and Cth2 are zinc-finger proteins that bind to specific AU-rich elements (ARE) located in the 3’-UTR of target mRNAs promoting the destabilization of these mRNA molecules [17–19]. The Cth1 and Cth2 targets include mRNAs for Fe-consuming processes, which in this way are downregulated under Fe scarcity. Both Cth1 and Cth2 proteins are functionally redundant in part, although Cth1 acts at early stages upon Fe deprivation and Cth2 acts in a time-sustained way [17,18]. The low number of Cth1-specific targets includes mRNAs for highly Fe-consuming mitochondrial respiration, while Cth2 induces degradation of a broader number of mRNA species encoding proteins involved in the tricarboxylic acids cycle, respiration, Fe-S cluster formation, heme biosynthesis or fatty acids metabolism. *CTH1* and *CTH2* mRNAs are as well targeted by both Cth1 and Cth2 proteins, resulting in an auto- and cross-regulatory loop that limits expression of the Cth1 and Cth2 proteins and causes their time-regulated expression [20]. Cth2 also post-transcriptionally controls dNTP levels, and therefore DNA metabolism and repair, by degrading *WTM1* mRNA, which encodes a protein that anchors the Fe-requiring ribonucleotide reductase enzyme at the nucleus [21]. Thus, in Fe-starvation conditions Cth2 facilitates nuclear export and accumulation of active ribonucleotide reductase complex at the cytosol.

The Fe regulon is also activated by other environmental stresses on *S*. *cerevisiae* cells [9]. One of these is oxidative stress, which transitorily provokes Aft1 internalization into the nucleus and transcriptional activation of the Aft1-targeted genes [22]. Transcript levels are additionally modulated along this response by their respective decay rates and thus, *FTR1* and *FET3* mRNA levels are maintained low upon oxidative stress by transcript destabilization through the general 5’–3’ mRNA decay pathway mediated by the Ccr4 deadenylase and the Xrn1 5’-3’-exonuclease [22]. Based on the prooxidant properties of the Ftr1/Fet3-mediated reductive pathway for Fe uptake, we proposed [22] that downregulation of *FTR1* and *FET3* mRNAs through increased molecule turnover would have a balancing effect under oxidative stress conditions. In yeast cells, stress induced by arsenate (which also acts as ROS generator) similarly causes downregulation of *FTR1* and *FET3* mRNA levels in a Ccr4- and Xrn1-dependent and Cth2-independent manner [23]. Although we have previously shown [22] that the modulation of *FTR1* and *FET3* mRNA stability under oxidative stress is independent of Cth2, we wanted to know whether or not Cth2 plays a regulatory role under oxidative stress at a genome-wide scale. In this work we demonstrate that upon oxidative stress induced by hydroperoxide Cth2 exerts a transitory regulation of a number of genes of the Fe regulon that contributes to the adaptation of *S*. *cerevisiae* cells in such stress conditions.

## Materials and Methods

### Strains, plasmids and growth conditions

*S*. *cerevisiae* W303-1A (*MAT***a** *ura3-1 ade2-1 leu2-3*,*112 trp1-1 his3-11*,*15*) was employed as wild type strain. The following mutants (described in Ref. 22) derive from it: MML1081 (*Δcth1*::*natMX Δcth2*::*kanMX4* [YIplac128]::*LEU2*); MML1082 (*Δcth1*::*natMX Δcth2*::*kanMX4* [pMM928]::*LEU2*); MML1114 (*Δcth1*::*natMX4 Δcth2*::*kanMX4* [YIplac211]::*URA3 tetO*_*2*_*-FTR1*::*kanMX4* [pCM244 (*tetR’-SSN6*)]::*LEU2*); MML1116 (*Δcth1*::*natMX4 Δcth2*::*kanMX4* [pMM942]::*URA3 tetO*_*2*_*-FTR1*::*kanMX4* [pCM244 (*tetR’-SSN6*)]::*LEU2*); and MML1196 (*Δxrn1*::*kanMX4*). Plasmids pMM928 and pMM942 are integrative plasmids derived from the vectors YIplac128 and YIplac211 respectively, and contain the *CTH2* open reading frame plus about 800 upstream and 300 downstream base pairs. Plasmids were integrated in the respective chromosomal *LEU2* or *URA3* locus after linearization by *EcoR*V digestion. Plasmid pRS416-GFP-CTH2 expresses GFP in frame after the *CTH2* start codon, resulting in a GFP-Cth2 fusion protein under the transcriptional control of the own *CTH2* promoter [19].

Cells were grown at 30°C, with shaking in the case of liquid cultures. YPD (1% yeast extract, 2% peptone, 2% glucose) was employed as rich medium. Synthetic complete SC medium [24] was also used, supplemented with 2% glucose and the specific auxotrophic requirements for each strain. For repressing expression from the *tetO* promoter, cells were grown continuously during at least 40 hours (equivalent to at least 15 generations) in the presence of doxycycline (5 μg/ml). The extracellular Fe chelator bathophenanthroline sulfonate (BPS, from Sigma) at 75 or 100 μM (final concentration) was added to create Fe-starvation conditions. Growth assays were carried out by spotting serial 1:10 dilutions of exponential cultures onto YPD plates containing the compounds indicated in each case, and recording growth after 2 days incubation at 30°C.

### Transcriptome analysis

Exponential cultures of MML1081 and MML1082 cells growing in YPD medium (Fe-replete conditions) were treated with 0.1 mM *tert-*butyl hydroperoxide (*t*-BOOH, from Sigma) for 45 or 90 min before processing of samples. RNA purification, first strand cDNA synthesis and labeling as well as microarray hybridizations were performed as previously described [25]. Arrays were printed at the Genomics and Bioinformatics Service, Autonomous University of Barcelona, in UltraGAPS slides (Corning). Probes are PCR amplification products of genomic DNA resuspended in 50% dimethyl sulfoxide. The arrays contain probes for ca. 5800 *S*. *cerevisiae* genes (including 56 snoRNAs and snRNAs). They are biased towards the 3’ end of the ORFs, ranging from 200 to 1,200 bp in length. In addition, we included probes targeting the presumed 3’ UTRs for about 500 duplicated genes. All probes were spotted three times at different locations in each array. The sequences of the oligonucleotides used to generate the probes are available upon request.

For half of the four independent experiments we labeled the time 0 min sample with Cy3, and the 45 and 90 min samples with Cy5. We swapped the dyes in the other half of the hybridizations. Microarrays were scanned with an Axon Genepix 4100A scanner (MDS Analytical Technologies), and data were generated with the Genepix Pro 6 software. We first carried out a visual QC to flag abnormally looking spots. Then, we applied an automatic QC filter to remove spots that had a signal-to-noise ratio smaller than three in both channels. We took the ratio of median intensities per spot as a representative value of relative amount of mRNA. Arrays were normalized by an intensity-dependent method (print-tip loess) implemented in the CARMAweb package on the web [26], under the assumption that most genes would not change their expression level. All arrays were scaled and the values for duplicated spots were merged by calculating the median. We next checked how consistent expression data were as a whole. For this, we removed all spots with missing values in any array, which left us with 2250 features. Then, all 16 arrays were hierarchically clustered in six different ways using two similarity metrics: correlation (uncentered) and euclidean distance, and three clustering methods: single, complete and average linkage. This was done with Cluster 3.0 [27]. Arrays clustered in two clearly separate groups, one with arrays containing the 45 min samples and another with the 90 min samples, irrespective of yeast strain source. This was not unexpected because the induced stress response would be predominant over the *CTH2* influence. There was, however, one 45 min-array and another 90 min-array that never clustered in the corresponding group, and they were hence removed from further studies.

To select for genes differentially expressed in the *Δcth2* strain as compared to the control *CTH2* strain, we analyzed the 45 min and 90 min time points separately. First, we only considered features with values from at least two arrays for each condition (strain and treatment time). Then the median value for each feature at a given condition was calculated. These medians were subtracted (as log_2_ ratios) between the *Δcth2* mutant and the control *CTH2* strain. For each time, genes with a difference higher than 1.5-fold (log_2_ = 0.585) increase in expression in the mutant were selected.

### Northern blot analyses

RNA isolation and electrophoresis, probe labeling with digoxigenin, hybridization, and signal detection were done as described previously [28]. Gene probes were generated by PCR from genomic DNA, using oligonucleotides designed to amplify internal open reading frame sequences. *SNR19* (U1 snRNA) was employed as loading control.

### Western blot analyses

Western blot analyses were done as already described [29]. Rabbit anti-GFP (IgG fraction, Molecular Probes) and anti-hexokinase 1 (US Biologicals) antibodies were used at 1:500 and 1:5000 dilutions respectively.

### Cell syncronization and cytometry analyses

Cells were synchronized in the G1 stage of the cell cycle by incubating exponentially growing cells in YPD medium at a concentration of 1x10^7^ cells/ml with 4 μg/ml α-factor (GenScript) during 45 min, followed by addition of the same amount of α-factor and an additional 45 min incubation. Cells were released from the G1 arrest by filtration and extensive washing with prewarmed (30°C) YPD medium, and resuspension in fresh medium at the original cell concentration. Samples were taken at different times and flow cytometry was performed following standard procedures. The percentage of budded cells was determined under the microscope in parallel fixed samples.

### Other methods

GFP-tagged proteins were visualized with an Olimpus BZ51 fluorescence microscope, after nuclear staining of cell samples with Hoechst (5 μg/ml, from Sigma) during 4 to 6 min. U-MNUA2 and U-MNUA3 filters were employed respectively for Hoechst and GFP staining. Intracellular Fe was determined as described previously [30], with samples of 2x10^7^ cells.

## Results

### Cth2 modulates the mRNA levels of genes of the Fe regulon during the response to hydroperoxide stress

**Fig 1:**
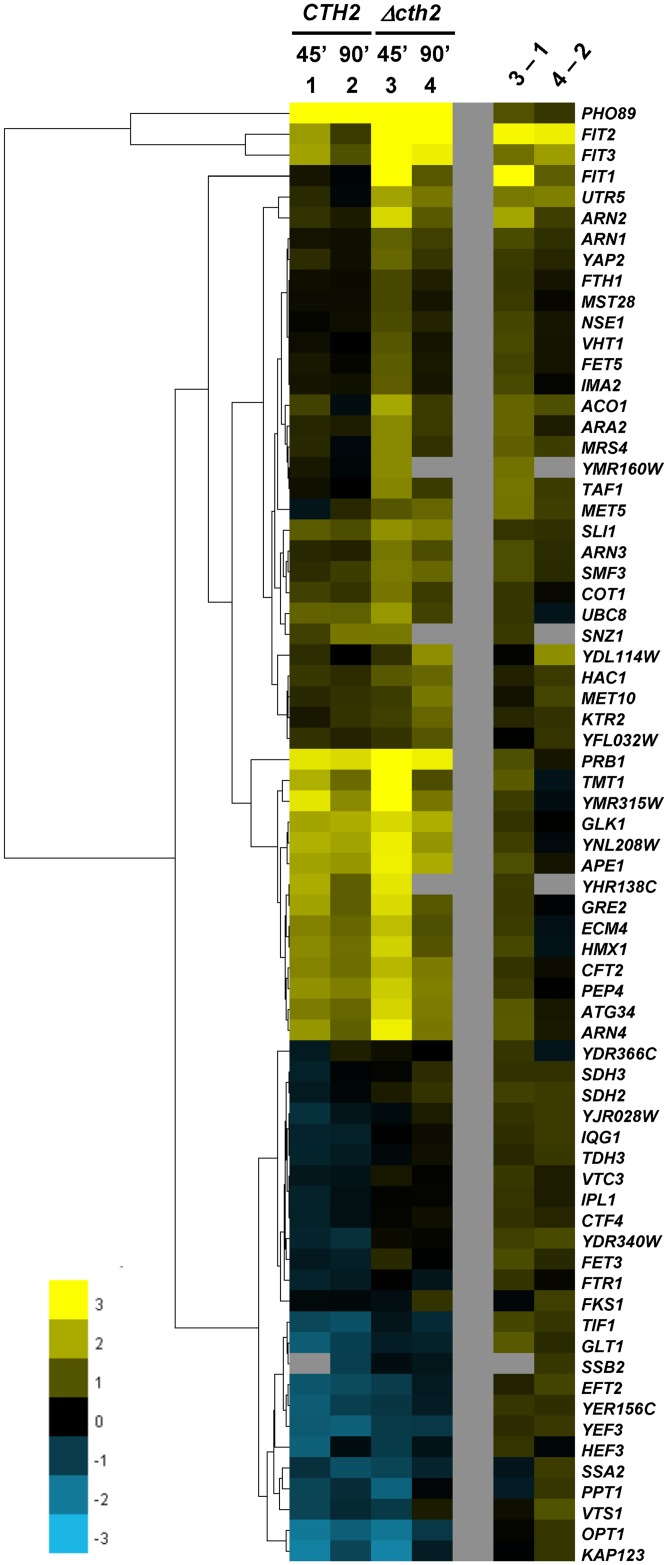
Set of genes upregulated in the absence of *CTH2* under oxidative stress conditions. The heat map shows the relative expression levels upon 0.1 mM *t-*BOOH treatment (ratio time 45 min or 90 min vs time 0) of the 70 genes that showed upregulated expression in the *Δcth2* mutant (MML1081) as compared to the strain expressing *CTH2* (MML1082). To identify patterns of expression we performed cluster analysis with a hierarchical method implemented in the cluster 3.0 software. The tree obtained with this method is shown on the left of the heat map. Three distinct patterns are observed (see text). The differential expression between the *Δcth2* and the *CTH2* strains (3–1 and 4–2) is also shown. Data depicted are log_2_ values and are the median for three or four microarrays. Upregulation is shown in yellow; downregulation in blue. Raw experimental data are deposited at ArrayExpress database, with accession number E-MTAB-4122.

To address the possible function of Cth2 in the oxidative stress response, cultures in rich YPD medium of two *S*. *cerevisiae* strains respectively containing the wild type *CTH2* gene or the null *Δcth2* mutation and both of them lacking the *CTH1* gene were subjected to transcriptomic analysis upon treatment with the oxidant *t*-BOOH for 45 and 90 min at 0.1 mM concentration. We have previously shown that these experimental conditions induce expression of diverse genes indicative of generation of an oxidative stress situation [3]. Considering the mRNA destabilizing function of Cth2, we focused our attention on transcripts that displayed higher levels in the *Δcth2* mutant compared to the *CTH2* control strain during the response to *t-*BOOH treatment, using as reference the samples from untreated cultures. Seventy genes had upregulated expression (more than 1.5-fold) in the *Δcth2* mutant compared to the wild type in at least one of the treatment time points (Fig 1 and S1 Table). Of these, sixteen become also upregulated in the mutant during Fe starvation [17,18], including six members of the Fe regulon: *FIT1*, *FTH1*, *VHT1*, *MRS4*, *HMX1* and *ARN4* (Table 1). Eight additional members of this regulon that had not been described as Cth2-regulated upon Fe starvation, however, display higher transcript levels in the absence of Cth2 during the *t*-BOOH stress response (Table 1). Besides the Fe homeostasis-related genes, no other GO (Gene Ontology) functional categories were enriched among the genes listed in Table 1.

**Table 1:** Genes whose mRNA levels upon oxidative stress are dependent on the presence of Cth2^a^.

Gene^b^	Aft1/Aft2 regulon^c^	AREs^d^	Gene^b^	Aft1/Aft2 regulon^c^	AREs^d^
*PHO89*		416	*YNL208W*		
*FIT2*	Yes	52, 306	***APE1***		478
*FIT3*	Yes	238	*YHR138C*		
***FIT1***	Yes	264	*GRE2*		
*UTR5*		320	*ECM4*		
*ARN2*	Yes		***HMX1***	Yes	
*ARN1*	Yes		*CFT2*		
***YAP2***			***PEP4***		
***FTH1***	Yes		***ATG34***		52
*MST28*		404, 419	***ARN4***	Yes	
*NSE1*			*YDR366C*		
***VHT1***	Yes		***SDH3***		91
*FET5*			***SDH2***		163, 309, 328
*IMA2*			*YJR028W*		
***ACO1***		33, 151, 178	*IQG1*		
*ARA2*		327	*TDH3*		
***MRS4***	Yes		*VTC3*		
*YMR160W*			*IPL1*		
*TAF1*			*CTF4*		
*MET5*		300, 348	*YDR340W*		
*SLI1*			*FET3*	Yes	
*ARN3*	Yes		*FTR1*	Yes	
*SMF3*	Yes	265	*FKS1*		
*COT1*			*TIF1*		
*UBC8*			***GLT1***		15, 37, 45, 53, 61, 69, 77
*SNZ1*			*SSB2*		
*YDL114W*			*EFT2*		
***HAC1***			***YER156C***		
*MET10*		354, 472	*YEF3*		
*KTR2*			*HEF3*		
*YFL032W*			*SSA2*		357
*PRB1*		215	*PPT1*		
*TMT1*			*VTS1*		
*YMR315W*			*OPT1*		
*GLK1*			*KAP123*		

^a^ From data in Fig 1 and S1 Table. See Materials and Methods for experimental conditions

^b^ Genes marked in bold become also upregulated in *Δcth2* cells in Fe-starvation conditions [17, 18]

^c^ From Ref. 8

^d^ Putative 3’ARE elements (5’-UAUUUAUU-3’ or 5’-UUAUUUAU-3’) located within 500 nucleotides after the stop codon. Data from Ref. 18 or from our analyses using the Regulatory Sequence Analysis Tools (RSAT)

A clustering analysis was applied to the genes expressing higher mRNA levels in the Cth2-deficient cells in the oxidative stress conditions. It resulted in basically three groups of genes (Fig 1): (i) some of them were not, or only modestly, induced upon *t*-BOOH treatment in the *CTH2* cells, while induction was prominent in the *Δcth2* mutant; (ii) other genes were considerably induced in both strains, although at a larger extent in the mutant; and (iii) finally other genes became downregulated upon *t*-BOOH treatment at a larger extent in the wild type than in the *Δcth2* mutant strain. In most cases the response was more intense at min 45 than at min 90. Most of the genes of the Fe regulon identified in this study belong to the first group. However, *FET3* and *FTR1* (which belong to the third group) are exceptions (Fig 1). Nevertheless, the differences in the mRNA levels of both genes between *CTH2* and *Δcth2* cells are subtle, which is in agreement with our previous results [22] showing that stability of the respective mRNAs does not depend on Cth2.

The presence of ARE motifs in the 3’-UTR region of the listed genes was analyzed (Table 1). Some of them have been described previously [17,18]. However, some other genes differentially expressed in the *CTH2* and *Δcth2* cells during the *t*-BOOH treatment but not identified previously in the Fe starvation stress conditions also contain canonical 3’-UTR ARE motifs. Remarkably, these include three genes of the Aft1/Aft2 regulon: *FIT2*, *FIT3* and *SMF3*. Overall, nineteen of the seventy genes characterized as Cth2-modulated at the mRNA level in the present study contain canonical ARE motifs (Table 1). Presumably, the other genes in the list that do not contain such canonical motifs may contain cryptic motifs or be indirectly regulated by Cth2. This same situation has been described by Puig et al. [18] in the response to Fe starvation conditions.

**Fig 2:**
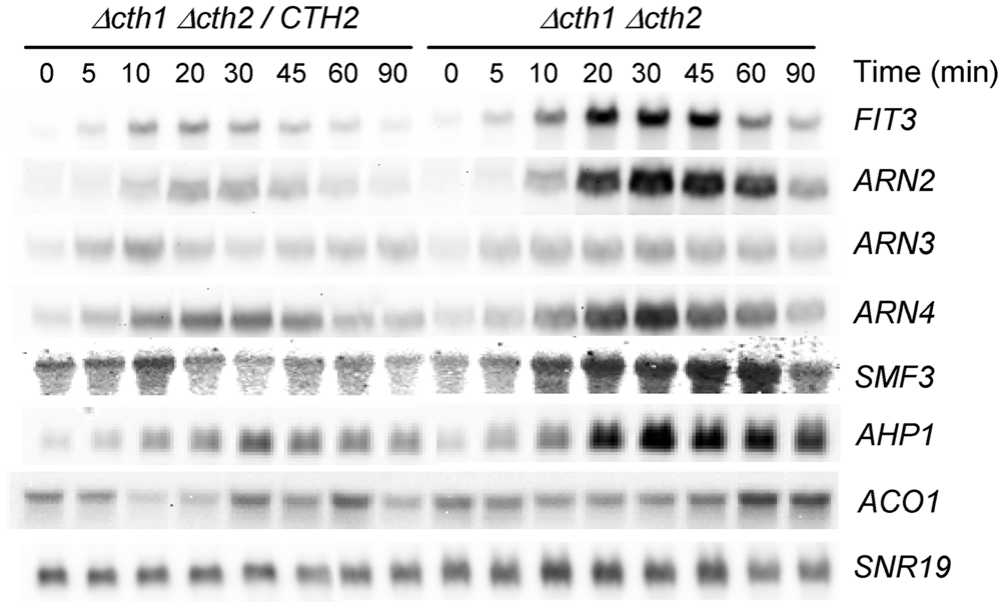
Northern blot analysis of gene expression under oxidative stress conditions. Exponentially growing cells of strains MML1082 (*Δcth1 Δcth2 / CTH2*) and MML1081 (*Δcth1 Δcth2*) in YPD medium at 30°C were treated with 0.1 mM *t*-BOOH for different time periods, before sample preparation and hybridization with the indicated gene probes. *SNR19* was employed as loading control.

We confirmed our genome-wide results for several Aft1/Aft2 regulon genes by Northern blot analysis (Fig 2). Indeed, we observed that during the stress response the upregulation of the genes tested was exacerbated in the absence of Cth2, with maximum levels between min 30 and 45. In parallel, *ACO1*, a gene whose mRNA levels are also modulated by Cth2 in Fe starvation conditions [17] displayed sustained upregulated mRNA levels as well upon *t*-BOOH treatment in the *Δcth2* cells. We also included in this study *AHP1*, a peroxiredoxin-encoding gene that responds to oxidative stress by upregulating its expression [31]. Although it did not pass our filtering conditions for the microarray data, it shows a clear modulation by Cth2 in our experimental conditions (Fig 2). *AHP1* contains two canonical 3’-UTR ARE motifs at positions 256 and 365.

### Cth2 protein levels increase upon hydroperoxide treatment

**Fig 3:**
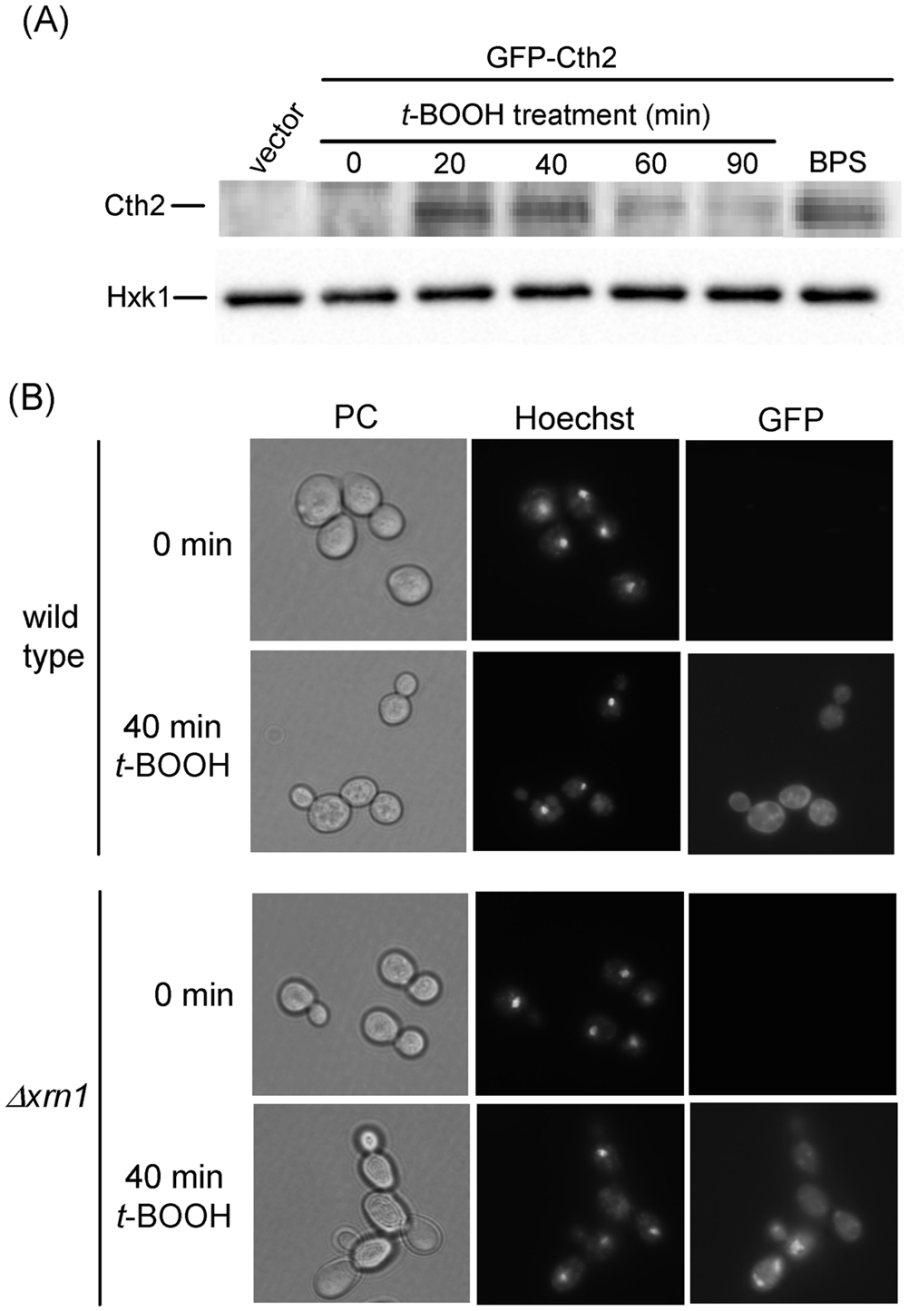
Expression of the Cth2 protein upon oxidative stress. (A) Western blot analysis of GFP-Cth2 levels in MML1081 (*Δcth1 Δcth2*) cells transformed with plasmid pRS416-GFP-CTH2 or with vector pRS416, growing exponentially in SC medium at 30°C. Cultures were treated with 0.4 mM *t*-BOOH for the indicated times or with 100 μM BPS for 6 hours. Anti-GFP antibody was employed for western blot of protein extracts (upper panel), and anti-hexokinase 1 (Hxk1) was employed as loading control (lower panel). (B) Fluorescence microscopy of W303-1A (wild type) or MML1186 (*Δxrn1*) cells transformed with pRS416-GFP-CTH2, growing exponentially in SC medium at 30°C and then treated for the indicated times with 0.4 mM *t*-BOOH. Prior to GFP fluorescence analysis samples were stained with Hoechst for nuclei localization. The corresponding phase contrast fields (PC) are also shown.

Our above results fit the previously described observation that the absence of Cth2 promotes *FIT3* mRNA stabilization in oxidative stress conditions, and that expression of the *CTH2* gene is upregulated in response to oxidative stress [22]. In accordance, transitory accumulation of the Cth2 protein occurs in *t*-BOOH treated cells (Fig 3A), paralleling the reported effects on the levels of mRNAs of some genes. We also determined the intracellular location of Cth2 upon the oxidant treatment, using a GFP-Cth2 derivative expressed under the own *CTH2* promoter. As it occurs upon Fe starvation [19], during the oxidative stress Cth2 remained cytoplasmically localized, although some concentrated foci were also observable (Fig 3B). By contrast, no fluorescence was observed in untreated cells. In Fe-starved cells, Cth2 localizes at P-bodies, where it carries out its mRNA degradation functions. Such localization is more clearly manifested in *Δxrn1* cells, which are compromised in the Xrn1-mediated 5′ to 3′pathway for mRNA decay. In the Xrn1-less mutant under Fe-starved conditions, a GFP-Cth2 fusion protein displays a punctuated location characteristic of P-bodies and colocalizes with P-body markers [19]. Hence, we analyzed the location of the GFP-Cth2 construct in *Δxrn1* cells upon *t*-BOOH treatment. In many cells we also observed clear fluorescent foci at 40 min of treatment (Fig 3B), which disappeared at later times (not shown). This is therefore suggestive of an active role of Cth2 in degradation of some mRNA molecules at P-bodies immediately after application of an oxidative stress.

### The absence of Cth2 causes cell growth retardation upon hydroperoxide treatment

**Fig 4:**
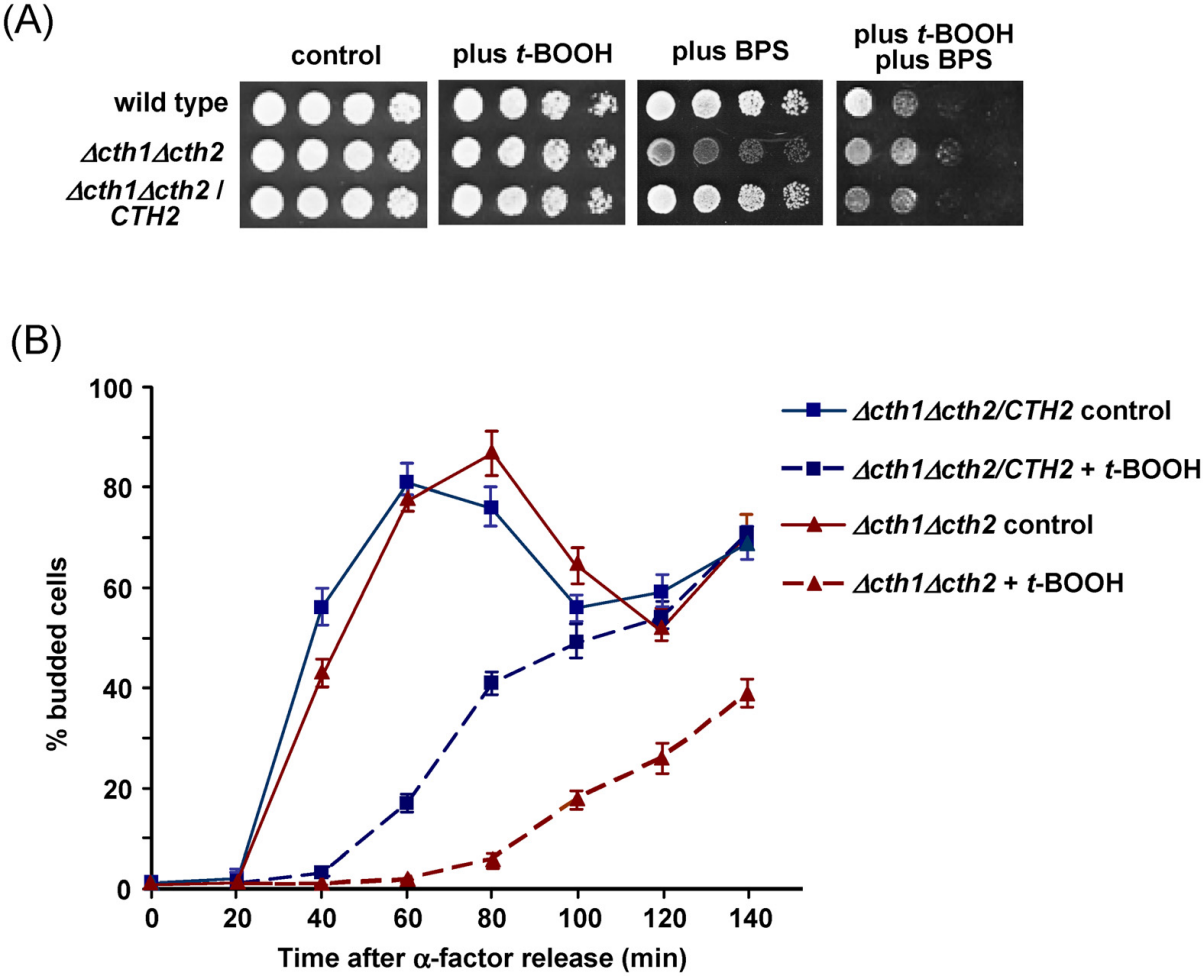
Sensitivity of *CTH2* and *Δcth2* mutant cells to oxidative stress. (A) Growth assays of serial dilutions of W303-1A (wild type), MML1081 (*Δcth1 Δcth2*) and MML1082 (*Δcth1 Δcth2* / *CTH2*) cells on YPD plates without additions (control) or with *t-*BOOH (0.1 mM) and/or BPS (75 μM). (B) Percentage of budded cells in cultures of *Δcth1 Δcth2* / *CTH2* and *Δcth1 Δcth2* strains released from α-factor arrest (time 0) in YPD medium without (control, continuous lines) or with 0.3 mM *t*-BOOH treatment (dashed lines). The mean (± s.d.) of three independent experiments is shown.

Cells lacking Cth2 are not hypersensitive to 0.1 mM *t*-BOOH in plate growth assays when compared to cells expressing *CTH2* (Fig 4A). Increasing the oxidant concentration did not result in a hypersensitivity phenotype of *Δcth2* cells in the plate assays (not shown). However, since the Cth2-mediated effects on mRNA levels described above seem to occur shortly after application of the oxidative stress, we reasoned that the biological role of Cth2 could be limited to the initial stages of the applied oxidative stress conditions. Previous studies [32–34] showed that oxidative stress delays exit from G1 cell cycle arrest in yeast. Consequently, we analyzed how the absence of Cth2 could affect the ability of α factor-mediated G1-arrested cells to exit from such arrest in the presence of *t*-BOOH. Since initiation of bud formation in yeast cells at G1 parallels entry into S phase, initially we employed the percentage of budded cells as a marker of the cell cycle stage. Thus, we observed that the oxidant causes a delay in the release from G1 compared to untreated cells even in wild type conditions. However, in the *Δcth2* mutant this delay is considerably longer than in cells expressing Cth2 (Fig 4B). These results were confirmed by the determination of the ratio of 1n and 2n cells through FACS analyses (S1 Fig). In the absence of the oxidative stress *Δcth2* cells do not display any significant defect concerning the release from the G1 arrest, which is in agreement with the fact that the ratio between 1n and 2n cells is very similar in *CTH2* and *Δcth2* cells during exponential growth (S1 Fig). This supports the absence of constitutive cell cycle defects in the mutant.

**Fig 5:**
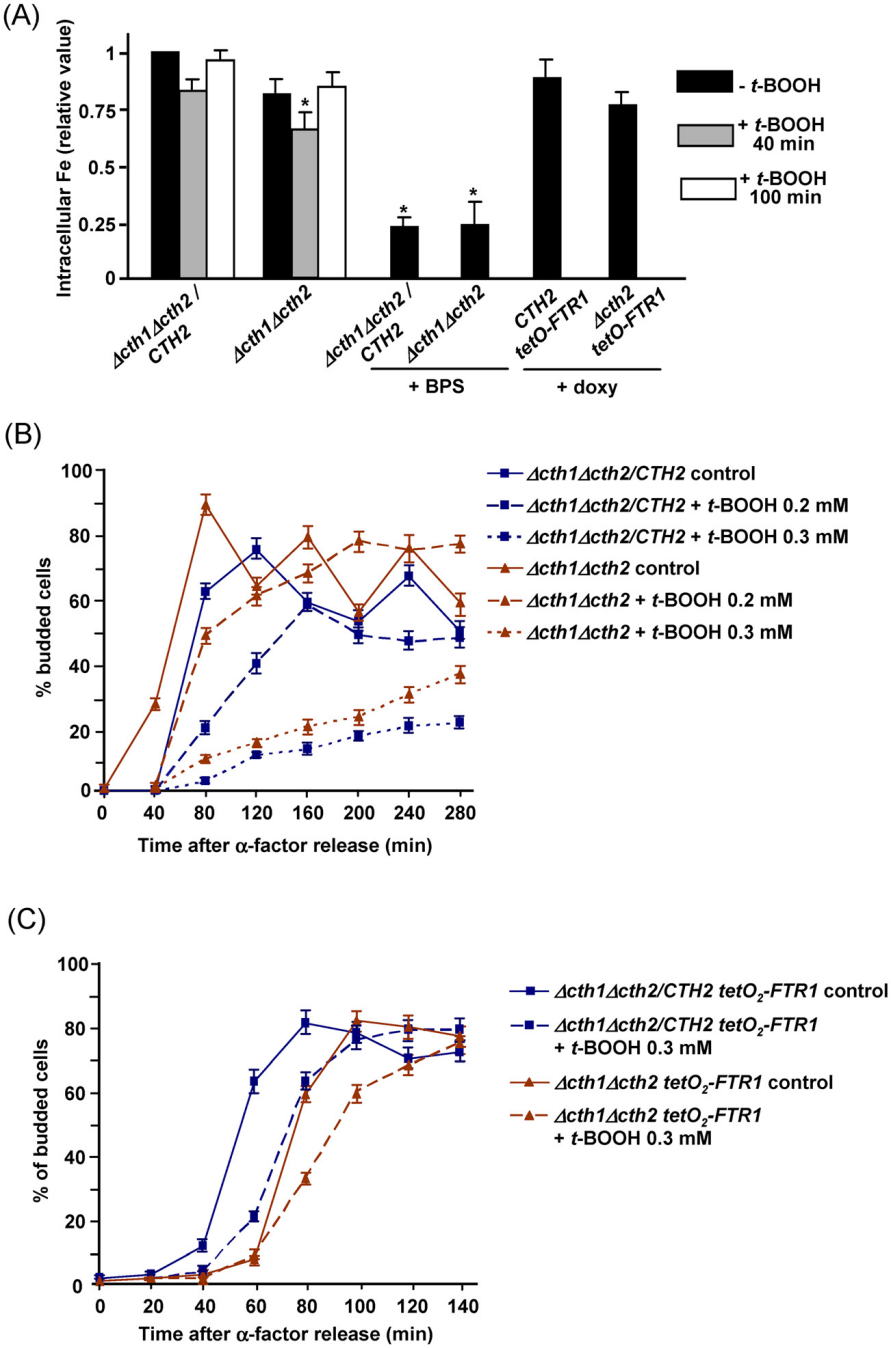
Sensitivity of *CTH2* and *Δcth2* mutant cells to oxidative stress under iron limitation conditions. (A) Intracellular Fe levels in the following strains: MML1082 (*Δcth1 Δcth2* / *CTH2*) and MML1081 (*Δcth1 Δcth2*) growing in liquid YPD medium without or with *t*-BOOH (0.3 mM, indicated times), and without or with BPS (75 μM, 16 hours); or MML1116 (*Δcth1 Δcth2* / *CTH2 tetO*_*2*_*-FTR1*) and MML1114 (*Δcth1 Δcth2 tetO*_*2*_*-FTR1*) growing in liquid YPD medium with doxycycline (5 μg/ml, 40 hours). Bars represent the mean of three experiments (± s.d.), and are normalized with respect to *Δcth1 Δcth2* / *CTH2* cells without additions, which are given the unit value. These are also employed as reference to analyze the statistical significance of the value differences (Tukey-Kramer test, *: p<0.05). (B) Percentage of budded cells in cultures of *Δcth1 Δcth2* / *CTH2* and *Δcth1 Δcth2* strains grown during 16 hours in YPD medium plus 75 μM BPS, arrested with α-factor in this same medium and then synchronously released at time 0 in BPS-containing YPD medium without (control, continuous lines) or with 0.2 or 0.3 mM *t*-BOOH (dashed lines). (C) Percentage of budded cells in cultures of *Δcth1 Δcth2* / *CTH2 tetO*_*2*_*-FTR1* and *Δcth1 Δcth2 tetO*_*2*_*-FTR1* strains grown during 40 hours in YPD medium plus 5 μg/ml doxycycline, arrested with α-factor in this same medium and then synchronously released at time 0 in doxycycline-containing YPD medium without (control, continuous lines) or with 0.3 mM *t*-BOOH (dashed lines). In both (B) and (C) the mean (± s.d.) of three independent experiments is shown.

In steady state growth conditions Cth2-less cells do not show significant differences in intracellular Fe levels compared to wild type cells (Fig 5A), in accordance to the fact that basal expression levels of genes of the Fe regulon do not apparently differ between both strains in such non-stress conditions (Fig 2). We reasoned that the transitory cell cycle delay in Cth2-less cells under the oxidant treatment could be due to the observed increased upregulation of expression of diverse Fe uptake genes in the mutant compared to *CTH2* control cells. Upon oxidative stress yeast cells finely tune expression of the Aft1 regulon genes specifically involved in the reductive pathway for high affinity Fe uptake, in order to avoid excessive uptake through this pathway, which could involve generation of potentially toxic ROS such as hydroxyl radicals [8,11,22]. Abnormal upregulation of other Fe uptake genes such as *FIT1-3* could counteract the mentioned adaptative downregulation of the reductive pathway, for instance by providing excessive Fe to the Ftr1/Fet3 complex. To test the mentioned hypothesis, both *CTH2* and *Δcth2* cells were grown in the presence of a sub-inhibitory concentration of the Fe chelator BPS to produce partial Fe deprivation in the medium and the consequent decreased uptake. In these conditions, intracellular Fe levels were about 25% of those in untreated cells (Fig 5A). The BPS-treated cultures were arrested in G1 with α-factor, then released under *t*-BOOH treatment as above, with continuous presence of the Fe chelator, and at successive times the percentage of budded cells (Fig 5B) and the ratio between 1n and 2n cells (S2 Fig, part A) were determined. Release rate from the G1 arrest was *t*-BOOH dose-dependent, although in all cases such release was not delayed in the *Δcth2* mutant compared to the *CTH2* control. On the contrary, the mutant exhibited a moderately higher rate of cell cycle resumption than the *CTH2* cells. Therefore, Fe scarcity conditions rescue the temporary hypersensitivity of G1-arrested *Δcth2* cells to the hydroperoxide stress. This is in accordance with the pattern observed in plate growth assays where contrary to *CTH2* cells, the *Δcth2* mutant does not display increased growth defects in *t*-BOOH plus BPS-treated cells as compared with cells treated with BPS alone (Fig 4A).

To confirm the influence of the reductive pathway of the high affinity Fe transport system on the sensitivity to *t*-BOOH, we compromised the Ftr1/Fet3 transport complex by inactivating the *tetO*-controlled *FTR1* gene in both the *CTH2* and *Δcth2* genetic backgrounds. In these genetic conditions, long exposure to doxycycline results in complete inhibition of *FTR1* expression and consequent Ftr1 depletion, therefore compromising Fe uptake through the plasma membrane mediated by the reductive pathway [22]. This did not result in a statistically significant reduction of intracellular Fe compared to cells expressing Ftr1 (Fig 5A), probably because of Fe entry through the low affinity transport system [35]. In these Ftr1-less conditions *t*-BOOH also caused a delay in exit from G1 arrest compared to cells not treated with the oxidant, using both the percentage of budded cells (Fig 5C) and the 1n/2n cells (S2 Fig, part B) as markers of the cell cycle stage. However, the *t*-BOOH induced delay in the *Δcth2* mutant was only 20 min longer than in *CTH2* cells, compared with the delay of 60–80 min observed when *FTR1* was normally expressed (see Fig 4B). This therefore confirms that Fe entry through the Ftr1/Fet3 complex (maybe by excessive Fe provision from the Fit1-3 mannoproteins) is mostly responsible of the transitory inhibitory effect of *t-*BOOH on *Δcth2* cells.

Fe entrance through Ftr1/Fit3 in external oxidative stress conditions could provoke generation of hydroxyl radicals, with consequent damage of the own transport complex. This could explain the modest temporary decrease in intracellular Fe (about 18%) observed in wild type cells upon *t*-BOOH treatment, peaking at min 40–60 of treatment [22], and possibly reflecting temporary inhibitory effects on Fe uptake. Consequently, in the present study we compared intracellular Fe levels in *CTH2* and *Δcth2* cells upon 40 and 100 min of *t*-BOOH treatment with those levels in untreated control cells (Fig 5A). Remarkably, Fe levels in 40 min-treated *Δcth2* cells were statistically significantly lower compared to the control, supporting transitory occurrence of physiologically relevant damage on the Fe uptake machinery.

## Discussion

Cth2 plays a role in modulation of a set of mRNAs in Fe-scarcity conditions in order to optimize Fe metabolism in such stress situations, with some overlapping role played by Cth1 [17,18,20]. Cth2 also promotes metabolic remodeling in cells lacking frataxin protein [36]. Yeast Yfh1 frataxin is involved in intracellular Fe metabolism, including formation of Fe-S clusters at mitochondria. To avoid interference with Cth1-mediated effects, the present study, which addresses the function of Cth2 in oxidative stress conditions, has been done in null *Δcth1* mutants. We have shown that immediately after provoking an oxidative stress situation by *t*-BOOH the levels of a group of mRNAs are also modulated in a Cth2-mediated way. In some cases, the 3’-UTR region of the Cth2-downregulated mRNAs contains ARE motifs, while in others the regulation might be indirect or due to cryptic promoter elements in such oxidant conditions. Among the transcripts that are more intensely upregulated in the absence of Cth2 upon *t*-BOOH treatment there are a significant number of members of the Fe regulon, although mRNAs specific of the reductive pathway of the high-affinity Fe uptake are not included in this group. Some of the genes upregulated in Cth2-less cells in the present study are also regulated through Cth2 under Fe scarcity [17,18], but none of them was reported to change in a Cth2-dependent manner in Yfh1 frataxin-less cells [36]. We have described previously [22] that oxidative stress does not provoke immediate depletion of intracellular Fe levels, therefore arguing against Fe starvation as the cause of upregulation of the Aft1-regulated genes in our experimental conditions.

As occurs with other members of the Fe regulon, transcription rate and transcript levels of *CTH2* (itself a member of the regulon) increase upon oxidative stress by *t*-BOOH [22], and here we have shown that Cth2 protein levels also increase transitorily in such conditions. The fact that this protein may carry out a function in promoting decay of a subpopulation of mRNA molecules is supported by its transitory accumulation in P bodies-like structures, which becomes exacerbated when the decay machinery is compromised, for instance due to the absence of the Xrn1 exonuclease. By promoting the transitory decay of mRNAs of high-affinity Fe transport components common to the reductive and non-reductive pathways (FIT proteins) and also of those specific of the non-reductive pathway (ARN proteins), Cth2 would be controlling Fe uptake and perhaps also Fe partition among intracellular compartments, the latter function as well subject to regulation by Aft1 and Aft2. In aerobic conditions, the low affinity system for Fe uptake mediated by *FET4* [37] is less operative than the high affinity system, due to the signaling action of oxygen on *FET4* mediated by the transcriptional repressor Rox1 [37–39]. The ferrous iron transported by Ftr1/Fet3 is a potent generator of toxic hydroxyl radicals, which could provoke oxidative damage, among others on membrane lipids and on the own Ftr1/Fet3 complex. The presence of peroxides would maximize such damage [40]. To reduce those effects, yeast cells temporary control *FET3* and *FTR1* levels in a Cth2-independent manner immediately after becoming in contact with the oxidant [22]. Now we show an additional level of control of Fe uptake in oxidant conditions that is mediated by Cth2 and affects other components of the high affinity Fe uptake system. The physiological relevance of this control by Cth2 is manifested by the long delay (compared to wild type *CTH2* cells) in exit from G1 cell cycle arrest and in traversing the S phase in the *Δcth2* mutant in the presence of the hydroperoxide. Why yeast cells should modulate, through downregulation, the expression of other components of the uptake system in addition to the reductive steps? First, the Fe-binding Fit1-3 mannoproteins are upstream providers of potentially toxic Fe to the Ftr1/Fet3 complex. Second, the siderophore-bound ferric ions could be sent to the vacuole, where they could be reduced to the ferrous state and released again into the cytosol through Fet5 and Smf3 [8,11]. Interestingly, the transcript levels of these two vacuole membrane exporters of Fe are also controlled by Cth2 under hydroperoxide treatment (Table 1). In other words, transitory modulation of the non-reductive pathway by Cth2 can also be contemplated as an adaptation response to oxidant conditions. This modulation would not affect significantly to intracellular Fe levels in such conditions (Ref. 22, and Fig 5A), in agreement with the capacity of yeast cells to buffering the different intracellular Fe forms [41], but would avoid (or reduce) generation of toxic ROS, both at the plasma membrane and at the cytosol, in the overoxidizing environment.

The direct or indirect modulator role of Cth2 would not be limited to Fe homeostasis genes. Although not other GO categories appear overrepresented in the Cth2-regulated gene list of Table 1, some genes that participate at some level in the oxidative stress response display an increased upregulation in the absence of Cth2 upon the stress application. This is the case of the peroxiredoxin gene *AHP1*, and also of *ECM4*/*GTO2*, that encodes an Omega-class glutathione transferase that additionally has activity as peroxidase [42]. Among the genes that are differentially overexpressed in the *Δcth2* mutant 45 min after application of the *t*-BOOH treatment to return to wild type levels at min 90 (Fig 1) are also *PEP4*, encoding a vacuolar aspartyl protease required for degradation of oxidized proteins [43], and *GRE2*, encoding an oxidoreductase that participates in the response to osmotic and oxidative stress, among others [44]. *HMX1* also displays this same Cth2-modulated transcription pattern. Through its heme oxygenase activity, the Hmx1 protein participates in the regulation of Fe homeostasis as a member of the Fe regulon [8], but more recently it has been demonstrated that it also regulates the expression of several antioxidant genes, therefore relating Fe homeostasis with oxidant protection [45].

In conclusion, Cth2 would participate in the immediate response of yeast cells to oxidative stress conditions upon hydroperoxide treatment, by modulating the expression of diverse high affinity Fe uptake genes in order to minimize the potential oxidant effects of Fe transported through both the reductive and non-reductive pathways. At the same time, Cth2 would also limit upregulation of other genes during such adaptation response, which globally extends the scope of metabolic functions in which Cth2 is implicated. The results underscore the importance to regulate Fe homeostasis during the oxidative stress response to minimize potential oxidative damage.

## Supporting Information

S1 Fig Cells lacking Cth2 display cell cycle alteration upon oxidative stress. FACS analysis of MML1082 (*Δcth1 Δcth2* / *CTH2*) and MML1081 (*Δcth1 Δcth2*) cells released from α-factor arrest (time 0) in YPD medium without (control) or with 0.3 mM *t*-BOOH treatment. FACS of mid-exponential asynchronous cell cultures is also shown, with the position of the 1n and 2n peaks. Three independent experiments were done, and the cell distribution diagrams correspond to a representative one.
(PDF)

S2 Fig Fe deprivation rescues the cell cycle alterations occurring in cells lacking Cth2. (A) FACS analysis of MML1082 (*Δcth1 Δcth2* / *CTH2*) and MML1081 (*Δcth1 Δcth2*) cells grown during 16 hours in YPD medium plus 75 μM BPS, arrested with α-factor in this same medium and then synchronously released at time 0 in BPS-containing YPD medium without (control) or with 0.2 or 0.3 mM *t*-BOOH. FACS of mid-exponential asynchronous cell cultures in YPD plus BPS is also shown. (B) FACS analysis of MML1116 (*Δcth1 Δcth2* / *CTH2 tetO*_*2*_*-FTR1*) and MML1114 (*Δcth1 Δcth2 tetO*_*2*_*-FTR1*) cells grown during 40 hours in YPD medium plus 5 μg/ml doxycycline, arrested with α-factor in this same medium and then synchronously released at time 0 in doxycycline-containing YPD medium without (control) or with 0.3 mM *t*-BOOH. FACS of mid-exponential asynchronous cell cultures in YPD plus doxycycline is also shown. In both (A) and (B) three independent experiments were done, and the cell distribution diagrams correspond to a representative one.
(PDF)

S1 Table Expression levels of genes whose mRNA levels upon oxidative stress are dependent on *CTH2*. (XLS)
