# Supplementary figures and images for: Cth2 Protein Mediates Early Adaptation of Yeast Cells to Oxidative Stress Conditions
Source: PLoS One. 2016 Jan 29;11(1):e0148204. doi: 10.1371/journal.pone.0148204 (PMC4732752; doi:10.1371/journal.pone.0148204)

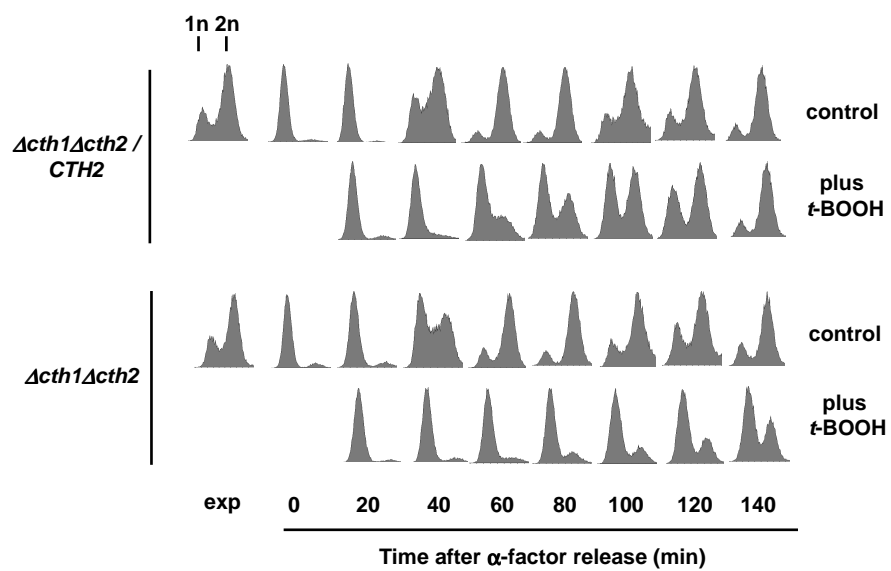

Supplement: S1 Fig — FACS analysis of MML1082 (Δcth1 Δcth2 / CTH2) and MML1081 (Δcth1 Δcth2) cells released from α-factor arrest (time 0) in YPD medium without (control) or with 0.3 mM t-BOOH treatment. FACS of mid-exponential asynchronous cell cultures is also shown, with the position of the 1n and 2n peaks. Three independent experiments were done, and the cell distribution diagrams correspond to a representative one. (PDF) [file pone.0148204.s001.pdf]

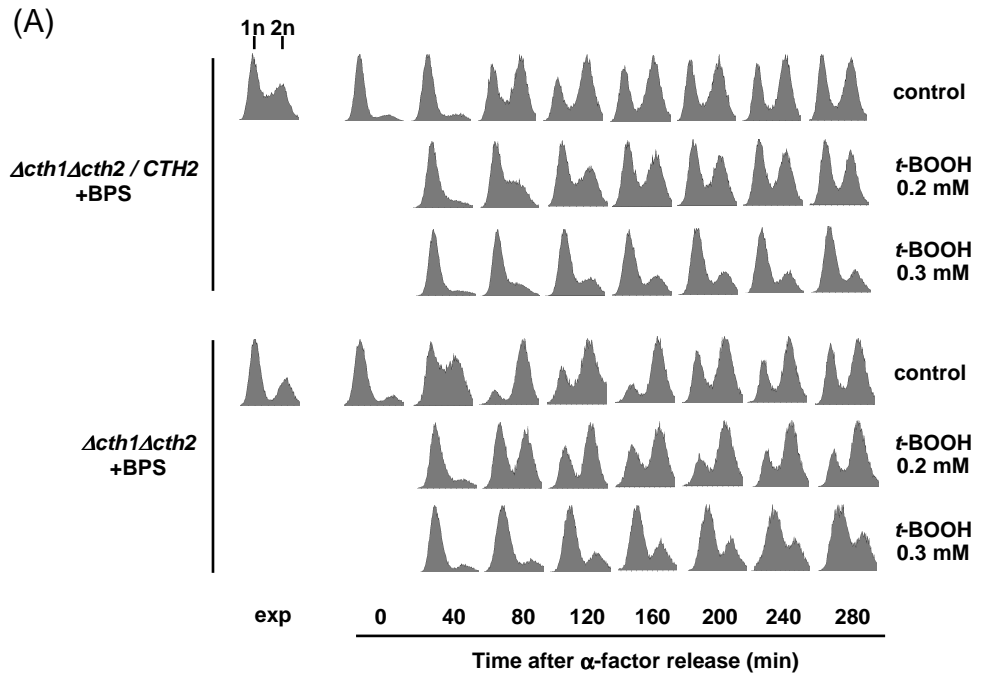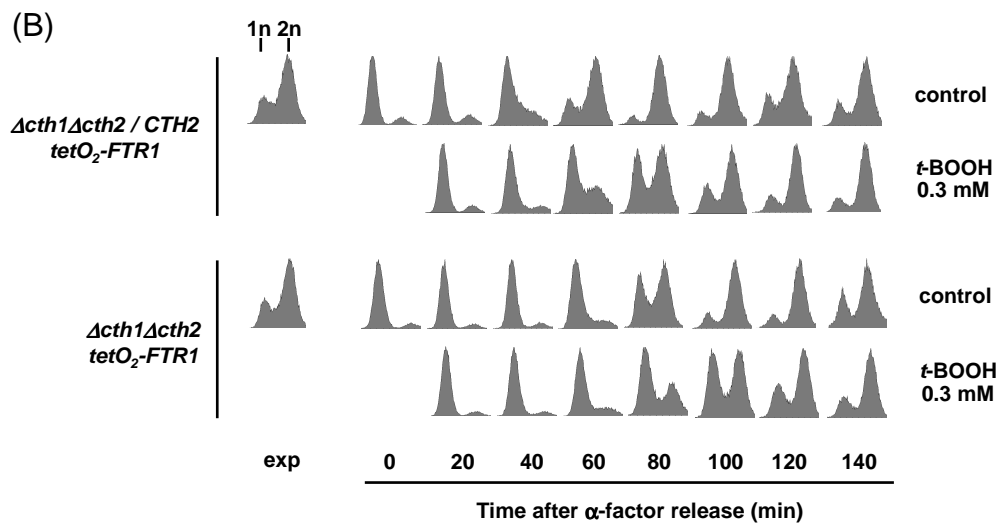

Supplement: S2 Fig — (A) FACS analysis of MML1082 (Δcth1 Δcth2 / CTH2) and MML1081 (Δcth1 Δcth2) cells grown during 16 hours in YPD medium plus 75 μM BPS, arrested with α-factor in this same medium and then synchronously released at time 0 in BPS-containing YPD medium without (control) or with 0.2 or 0.3 mM t-BOOH. FACS of mid-exponential asynchronous cell cultures in YPD plus BPS is also shown. (B) FACS analysis of MML1116 (Δcth1 Δcth2 / CTH2 tetO2-FTR1) and MML1114 (Δcth1 Δcth2 tetO2-FTR1) cells grown during 40 hours in YPD medium plus 5 μg/ml doxycycline, arrested with α-factor in this same medium and then synchronously released at time 0 in doxycycline-containing YPD medium without (control) or with 0.3 mM t-BOOH. FACS of mid-exponential asynchronous cell cultures in YPD plus doxycycline is also shown. In both (A) and (B) three independent experiments were done, and the cell distribution diagrams correspond to a representative one. (PDF) [file pone.0148204.s002.pdf]
